# PnBA-b-PNIPAM-b-PDMAEA Thermo-Responsive Triblock Terpolymers and Their Quaternized Analogs as Gene and Drug Delivery Vectors

**DOI:** 10.3390/polym13142361

**Published:** 2021-07-19

**Authors:** Athanasios Skandalis, Dimitrios Selianitis, Stergios Pispas

**Affiliations:** Theoretical and Physical Chemistry Institute, National Hellenic Research Foundation, 48 Vassileos Constantinou Avenue, 11635 Athens, Greece; thanos.skan@gmail.com (A.S.); dselianitis@eie.gr (D.S.)

**Keywords:** ABC triblock terpolymers, stimuli-responsive polymers, polyplexes, drug delivery, bioimaging

## Abstract

In this work, the ability of thermo-responsive poly [butyl acrylate-b-N-isopropylacrylamide-b-2-(dimethylamino) ethyl acrylate] (PnBA-b-PNIPAM-b-PDMAEA) triblock terpolymer self-assemblies, as well as of their quaternized analogs (PnBA-b-PNIPAM-b-QPDMAEA), to form polyplexes with DNA through electrostatic interactions was examined. Terpolymer/DNA polyplexes were prepared in three different amine over phosphate group ratios (N/P), and linear DNA with a 2000 base pair length was used. In aqueous solutions, the terpolymers formed aggregates of micelles with mixed PNIPAM/(Q)PDMAEA coronas and PnBA cores. The PnBA-b-PNIPAM-b-PDMAEA terpolymers’ micellar aggregates were also examined as carriers for the model hydrophobic drug curcumin (CUR). The complexation ability of the terpolymer with DNA was studied by UV–Vis spectroscopy and fluorescence spectroscopy by investigating ethidium bromide quenching. Fluorescence was also used for the determination of the intrinsic fluorescence of the CUR-loaded micellar aggregates. The structural characteristics of the polyplexes and the CUR-loaded aggregates were investigated by dynamic and electrophoretic light scattering techniques. Polyplexes were found to structurally respond to changes in solution temperature and ionic strength, while the intrinsic fluorescence of encapsulated CUR was increased at temperatures above ambient.

## 1. Introduction

Gene therapy has gained significant scientific attention for the treatment of diseases that arise from genetic abnormalities [1,2,3]. The presence of an effective vector that will carry out the efficient delivery of the genetic material through the cell membrane is a prerequisite [1,4,5]. There have been reported two major categories of gene delivery vectors, the viral and the nonviral, each one with its respective advantages and disadvantages [4,6].

Viral-mediated gene delivery systems [2,7] include, among others, adenoviruses and retroviruses and exhibit high transfection efficiency. However, viral gene delivery systems demonstrate limited carrying capacity, immunogenicity toxicity and high cost. 

The above-mentioned limitations can be overcome by the utilization of synthetic, nonviral gene delivery vectors as an appealing alternative. Such vectors interact electrostatically with the DNA and must have the ability to condense the DNA, be not immunogenic and toxic, promote cellular uptake and protect DNA from degradation. Cationic polymers [8,9], along with lipids [10,11,12,13], have emerged as the most promising nonviral gene delivery vectors. The most used cationic polymers for gene delivery applications are polyethyleneimine (PEI) [3,14], poly(L-lysine) (PLL) [15,16], poly(ethylene glycol) bis (amine) [17,18], poly (2-dimethylamino ethyl methacrylate) (PDMAEMA) [19,20,21] and poly (2-dimethylamino ethyl acrylate) (PDMAEA) [22,23]. The complexes of cationic polymers with DNA are widely known as polyplexes.

Stimuli-responsive polymers are promising candidates for gene delivery applications, due to their unique ability to adapt to changes in their environment after the application of external stimuli [24,25]. Temperature-responsive polymers demonstrate changes in their conformation/hydration state upon the application of temperature variations. PNIPAM [26,27,28], probably the most famous and the most-well studied thermo-responsive polymer, exhibits hydrophilic behavior at room temperature and has a lower critical solution temperature (LCST) at approximately 32 °C, above which it becomes less hydrophilic. The biocompatibility, along with its LCST being close to the human body temperature, make PNIPAM an attractive polymer for gene delivery applications [29,30,31,32,33]. Upon heating above the LCST, the PNIPAM chains at the periphery of the polyplexes shrink, leading to increased protection of the DNA against enzymatic degradation. Moreover, the enhanced hydrophobicity without complete dehydration can result in improved transfection efficiency and cellular uptake [34,35,36].

Amphiphilic block copolymer micelles are widely used as nanocarriers for the encapsulation and delivery of hydrophobic drugs as they seek to avoid possible side effects, decrease drug degradation after administration and enhance the bioavailability of the drug [37,38,39]. Curcumin (CUR) is a hydrophobic polyphenol compound that has gained significant attention due to its low cytotoxicity, anticancer effects and anti-inflammatory properties [40,41,42,43].

The aim of this work was the physicochemical investigation of the capability of PnBA-b-PNIPAM-b-PDMAEA terpolymers and of their quaternized analogs, PnBA-b-PNIPAM-QPDMAEA, to form complexes with DNA. Furthermore, the potential of the initial PnBA-b-PNIPAM-b-PDMAEA terpolymers to be utilized as carriers for the encapsulation of the hydrophobic model drug CUR for potential bioimaging purposes (due to CUR’s intrinsic fluorescence) was also examined, making use of the hydrophobic interactions with PnBA blocks. The ABC-type triblocks are composed of one hydrophobic PnBA block, one temperature-responsive PNIPAM block and one weak cationic polyelectrolyte PDMAEA block, which is converted into a strong cationic polyelectrolyte with permanent positive charges after post-polymerization functionalization with methyl iodide. In aqueous solutions, the terpolymers self-assemble in spherical micelles with PnBA cores and mixed PNIPAM/(Q)PDMAEA coronas and exhibit aggregation behavior due to secondary interactions, which are weaker in the case of the quaternized terpolymers. Further aggregation takes place upon heating above the LCST of PNIPAM because of the change in its hydrophobicity and the shrinkage of its chains. The complexation of the terpolymers micellar aggregates with DNA is possible through electrostatic interactions between the positive charges of the (Q)PDMAEA and the negative charges of the DNA.

## 2. Materials and Methods

### 2.1. Materials

Deoxyribonucleic acid (DNA) sodium salt from salmon testes (∼2000 bp) was obtained from Sigma-Aldrich (Athens, Greece). Curcumin was obtained from Merck (Athens, Greece). Ethidium bromide, sodium chloride and all other reagents were obtained from Sigma-Aldrich and used as received.

### 2.2. Triblock Terpolymer Synthesis

PnBA-b-PNIPAM-b-PDMAEA triblock terpolymers were synthesized by sequential RAFT polymerization using AIBN as the radical initiator, 2-(dodecylthiocarbonothioylthio)-2-methylpropionic acid as the chain transfer agent and 1,4-dioxane as the polymerization solvent. The conversion of PnBA-b-PNIPAM-b-PDMAEA triblock terpolymers into PnBA-b-PNIPAM-b-QPDMAEA strong cationic polyelectrolytes was achieved by a typical quaternization reaction in THF, using CH_3_I as the quaternizing agent.

**Scheme 1:**
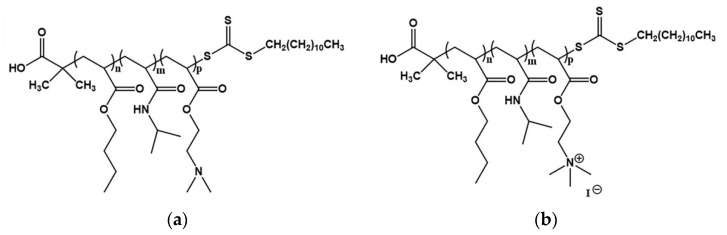
Chemical structure of (**a**) PnBA-b-PNIPAM-b-PDMAEA and (**b**) PnBA-b-PNIPAM-b-QPDMAEA triblock terpolymers.

More details about the synthetic procedure, the quaternization process, molecular characterization and aqueous solution properties of the terpolymers can be found in our previous work [44]. The chemical structures of PnBA-b-PNIPAM-b-PDMAEA and PnBA-b-PNIPAM-b-QPDMAEA triblock terpolymers are presented in Scheme 1a,b, respectively, and their molecular characteristics can be found in Table 1.

### 2.3. Polyplex Formation

The terpolymer/DNA polyplexes were formed by mixing terpolymer solution (5 × 10^−4^ g mL^−1^ in 0.01 M NaCl) and DNA solution (3.3 × 10^−4^ g mL^−1^ in 0.01 M NaCl) under gentle stirring at ambient conditions. The volume of the terpolymer solution was predefined and the corresponding volume of DNA solution was added for all N/P ratios formed. The final volume of the prepared mixed solutions was adjusted at 10 mL. The polyplexes were prepared in three different N/P ratios (0.5, 1.0 and 2.0).

### 2.4. Preparation of CUR-Loaded Micellar Aggregates

The procedure followed for the encapsulation of CUR into the triblock terpolymer nanostructures is described below. First, two separate stock solutions of the terpolymer and the drug were prepared in THF. The solutions were left to stand overnight to ensure the complete dissolution of the terpolymer and curcumin. Next, the two solutions were mixed in two different ratios and the mixtures were directly injected into distilled water under vigorous stirring until THF evaporation by heating at 65 °C.

### 2.5. Light Scattering

Dynamic light scattering (DLS) measurements were conducted on an ALV/CGS-3 compact goniometer system (ALVGmbH, Hessen, Germany), equipped with an ALV 5000/EPP multi-τ digital correlator with 288 channels and an ALV/LSE-5003 light scattering electronics unit for stepper motor drive and limit switch control. A JDS Uniphase 22 mW He-Ne laser (λ = 632.8 nm) was used as the light source. The solutions were filtered through 0.45 μm hydrophilic PTFE filters (Millex-LCR from Millipore, Billerica, MA, USA) before measurements. Each solution was measured five times at each angle and temperature and the average was used. The angular and temperature range for the measurements were 30–150° and 25–55 °C, respectively. The cumulants method and CONTIN software were utilized for the analysis of the obtained correlation functions. The size data and figures shown below are from measurements at 90°.

Electrophoretic light scattering (ELS) measurements were performed on a ZetaSizer Nano Series Nano-ZS (Malvern Instruments Ltd., Malvern, UK) equipped with a He-Ne laser beam at a wavelength of 633 nm and a fixed backscattering angle of 173°. Measurements were conducted at 25 °C and 45 °C. Data analysis was performed using the Henry correction of the Smoluchowski equation [45], after equilibration at 25 °C or 45 °C (depending on the measurement temperature). Reported zeta-potential (ζ_p_) values are the average of 50 runs.

### 2.6. UV–Vis

UV–V is measurements of the polyplexes were conducted on a Perkin Elmer (Lambda 19) UV–Vis-NIR spectrometer (Waltman, MA, USA) in a wavelength range of 200 to 400 nm. The polyplexes were measured at all three N/P ratios prepared.

### 2.7. Fluorescence Spectroscopy

#### 2.7.1. Ethidium Bromide Quenching Assay

The complexation ability of the terpolymers with DNA was investigated by studying the fluorescence of ethidium bromide (EtBr) in the whole N/P ratio range. A DNA solution (1 × 10^−4^ g mL^−1^) was initially prepared in 0.01 M NaCl, followed by the addition of ethidium bromide ([EtBr]/[P] = 4) to it. The EtBr-containing DNA solution was titrated with a concentrated terpolymer solution starting from N/P = 0 and ending at N/P = 8.0. Fluorescence spectroscopy measurements were carried out for the initial DNA/EtBr solution, as well as after each titration with the polymer solution. Fluorescence spectra were recorded on a Fluorolog-3 Jobin Yvon-Spex spectrofluorometer (model GL3–21). The excitation wavelength for the measurements was 535 nm and the emission was monitored at 600 nm.

#### 2.7.2. Fluorescence of CUR-Loaded Aggregates

The same instrument was used for fluorescence measurements of the CUR-loaded terpolymer aggregates and the excitation wavelength was set at 405 nm.

## 3. Results and Discussion

### 3.1. PDMAEMA-b-PNIPAM-b-(Q)PDMAEA/DNA Polyplexes

The ability of the nanostructures formed by PnBA-*b*-PNIPAM-*b*-PDMAEA and PnBA-*b*-PNIPAM-*b*-QPDMAEA triblock terpolymers in aqueous solutions to complex with DNA was investigated. The complexation is achieved through electrostatic interactions between the positively charged amine groups of the terpolymers (N) and the negatively charged phosphoric groups of the DNA (P). The terpolymer/DNA complex solutions were prepared in three different N/P ratios (N/P = 0.5, 1.0 and 2.0) using DNA with M_w_ = 2000 bp.

**Figure 1:**
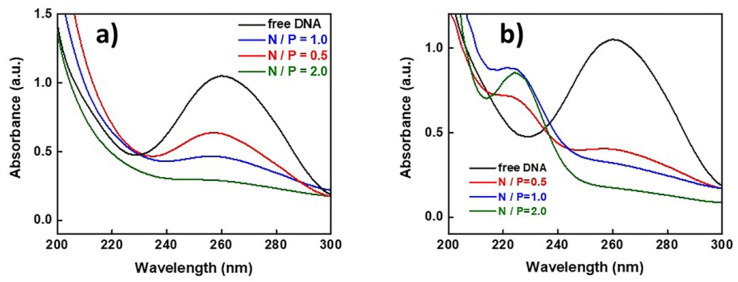
UV–Vis spectra of (**a**) PnBA_39_-*b*-PNIPAM_87_-*b*-PDMAEA_35_/DNA polyplexes and (**b**) PnBA_39_-*b*-PNIPAM_87_-*b*-QPDMAEA_35_/DNA polyplexes (spectrum of free DNA in black).

The successful formation of the polyplexes was confirmed by UV–Vis spectroscopy measurements. UV–Vis spectra for (a) PnBA-*b*-PNIPAM-*b*-PDMAEA/DNA and (b) PnBA-*b*-PNIPAM-*b*-QPDMAEA/DNA polyplexes are presented in Figure 1. The characteristic peak that corresponds to free DNA appears at 260 nm, while simultaneously a new peak is observed at 225 nm, which corresponds to the complexed DNA. However, this peak is only visible in the case of the quaternized terpolymers (Figure 1b). More specifically, for N/P = 0.5, both peaks are observed (at 225 and at 260 nm) and this can be attributed to the existence of both complexed and free DNA in the solution at the same time.

As the terpolymer concentration in the solution increases, the peak at 260 nm disappears and only the one at 260 nm is visible, meaning that the total amount of DNA participates in the formation of polyplexes. This occurs because the increase in the positive charges in the solution leads to the neutralization of the negative DNA charges.

On the other hand, in the case of PnBA-*b*-PNIPAM-*b*-PDMAEA/DNA polyplexes (Figure 1a), a similar decrease is observed in the height of the free DNA peak as the polymer concentration increases, but the peak at 225 nm that corresponds to the bonded DNA is not visible. This is an indication that the electrostatic interactions between terpolymer and DNA are significantly more intense when the quaternized terpolymers are used, due to the existence of permanent positive charges.

**Figure 2:**
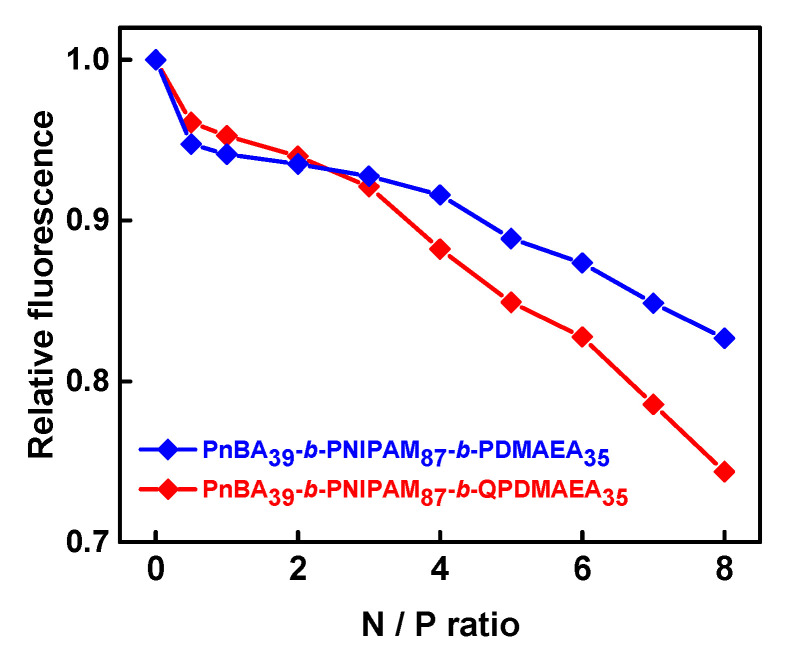
Ethidium bromide fluorescence quenching in PnBA_39_-*b*-PNIPAM_87_-*b*-PDMAEA_35_ (blue line) and PnBA_39_-*b*-PNIPAM_87_-*b*-QPDMAEA_35_ (red line) polyplexes with DNA.

Ethidium bromide fluorescence quenching assays (Figure 2) support the conclusion that the quaternized terpolymers interact more strongly and more efficiently with DNA molecules forming polyplexes. This is evident through the strongest decrease in relative fluorescence for the PnBA_39_-*b*-PNIPAM_87_-*b*-QPDMAEA_35_ polyplexes compared to PnBA_39_-*b*-PNIPAM_87_-*b*-PDMAEA_35_ ones, at N/P ratios greater than 3. However, even at an N/P ratio equal to 8, the relative fluorescence does not drop below 0.7, indicating that the terpolymer micellar aggregates do not interact sufficiently with DNA, possibly due to their aggregate structure, which hinders terpolymer/DNA interactions because of spatial constraints, including low access of amine/quaternary amine groups by DNA chains in the corona of the terpolymer micellar aggregates.

**Figure 3:**
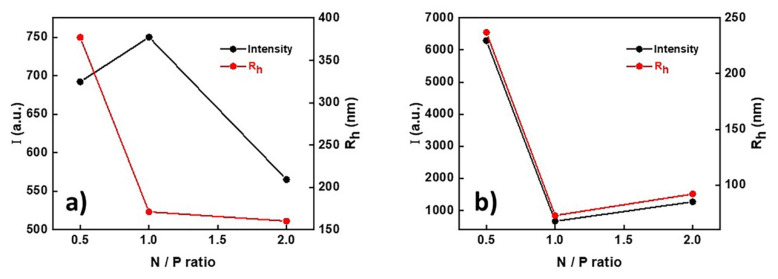
Variations in scattering intensity and *R_h_* with N/P ratio for (**a**) PnBA_39_-*b*-PNIPAM_87_-*b*-PDMAEA_35_/DNA and (**b**) PnBA_39_-*b*-PNIPAM_87_-*b*-QPDMAEA_35_/DNA polyplexes.

As depicted in Figure 3, the scattering intensity of PnBA_39_-*b*-PNIPAM_87_-*b*-PDMAEA_35_/DNA does not exhibit major changes for N/P = 0.5 and N/P = 1 ratios and decreases significantly for N/P = 2, showing that, in this case, particles with smaller mass are present. On the contrary, as far as the size of the polyplexes is concerned, it is observed that at N/P = 0.5, there are large aggregates (R_h_ = 380 nm approximately) and there is a great size decrease at N/P = 1 (R_h_ = 170 nm approximately). This rapid decrease in the size of the polyplexes is attributed to the formation of the smaller aggregates since the concentration of DNA increases and interactions are more efficient. At N/P = 0.5, the negatively charged phosphate groups are in excess and the formation of stable complexes with the polymeric nanostructures is more difficult. Therefore, the formation of aggregates is favored.

At the ratios where the amine groups of the terpolymer are either in equilibrium or in excess (the size of the polyplexes does not change at N/P = 2), it seems that the complexation with DNA is stronger and induces the breaking of the intermicellar aggregates and the formation of more stable structures.

At PnBA_39_-*b*-PNIPAM_87_-*b*-QPDMAEA_35_/DNA polyplexes, the scattering intensity shows a rapid decrease from N/P = 0.5 to N/P = 1 and remains practically unchanged for N/P = 2. As mentioned earlier, at N/P = 0.5, the DNA is in excess and the electrostatic interactions are weaker, leading to the formation of aggregates. The size of the polyplexes also shows a rapid decrease from 250 nm at N/P = 0.5 to 70 nm at N/P = 1. This decrease is attributed to the formation of smaller intermicellar aggregates in the solution.

At N/P = 2, there is a small increase in the size of the polyplexes (R_h_ = 95 nm). Moreover, it must be highlighted that the electrostatic interactions between the quaternized terpolymers and the DNA are much stronger than the interactions between the non-quaternized ones (amine form) with DNA, due to the existence of more positive charges.

Dynamic and electrophoretic light scattering were also utilized for more detailed investigations of the temperature effect on PnBA_39_-*b*-PNIPAM_87_-*b*-PDMAEA/DNA and PnBA-*b*-PNIPAM_87_-*b*-QPDMAEA/DNA polyplexes, because of the presence of the thermo-responsive PNIPAM block in the structure of the terpolymers.

**Figure 4:**
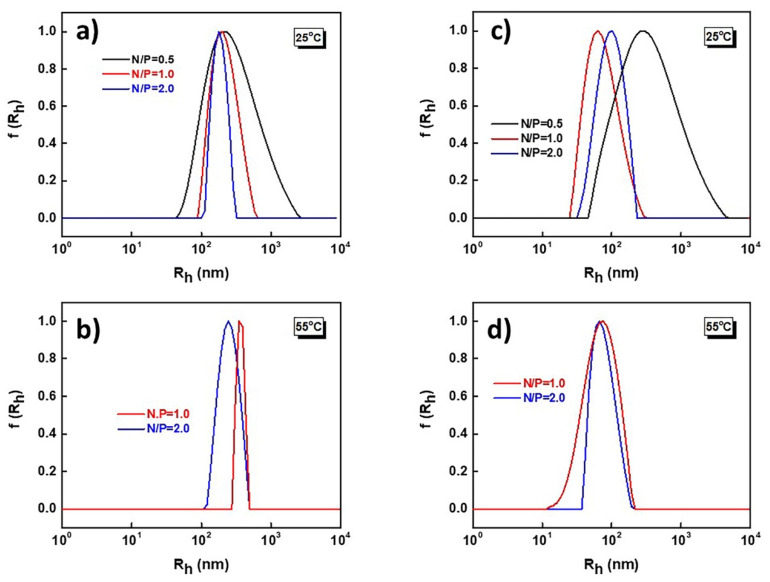
Size distributions of PnBA_39_-*b*-PNIPAM_87_-*b*-PDMAEA_35_/DNA (**a**,**b**) and PnBA_39_-*b*-PNIPAM_87_-*b*-QPDMAEA_35_/DNA (**c**,**d**) polyplexes at 25 °C (**a**,**c**) and at 55 °C (**b**,**d**).

The size distribution graphs from DLS measurements for PnBA_39_-*b*-PNIPAM_87_-*b*-PDMAEA35/DNA (a, b) and PnBA_39_-*b*-PNIPAM_87_-*b*-QPDMAEA_35_/DNA (c, d) polyplexes at 25 °C (a, c) and at 55 °C (b, d) are presented in Figure 4. Monomodal size distributions are observed in all cases. For PnBA_39_-*b*-PNIPAM_87_-*b*-PDMAEA_35_ /DNA polyplexes at 25 °C (Figure 4a), the size distribution becomes narrower as the polymer concentration in the solution increases. Similar behavior is observed for PnBA_39_-*b*-PNIPAM_87_-*b*-QPDMAEA_35_/DNA polyplexes at 25 °C (Figure 4c).

At 55 °C, precipitation phenomena took place for both complexes involving the non-quaternized and the quaternized terpolymers. For the other N/P ratios, the solutions remained stable, and the size distributions remained monomodal. In the case of PnBA_39_-*b*-PNIPAM_87_-*b*-PDMAEA_35_/DNA polyplexes (Figure 4b), it is also observed that the size distributions become narrower as the polymer concentration increases, while for PnBA_39_-*b*-PNIPAM_87_-*b*-QPDMAEA_35_/DNA polyplexes (Figure 4d), there are no significant changes.

**Figure 5:**
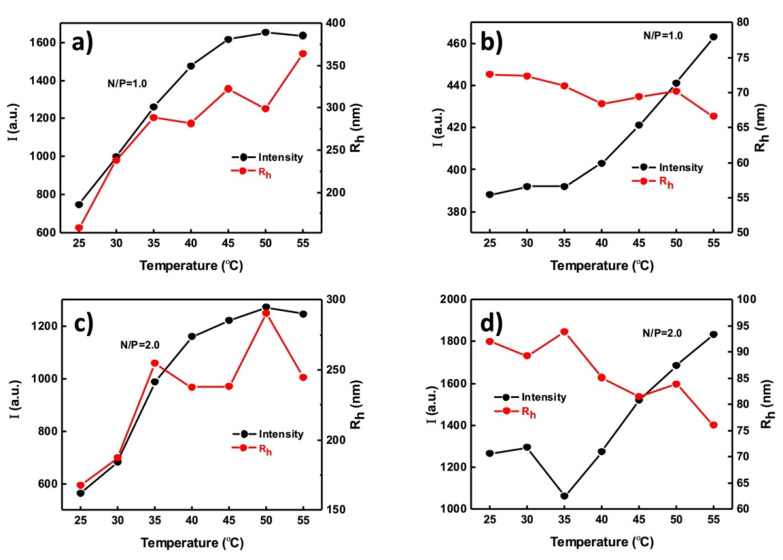
Scattering intensity and *R_h_* as a function of temperature for PnBA_39_-*b*-PNIPAM_87_-*b*-PDMAEA_35_ /DNA polyplexes at (**a**) N/P = 1.0 and (**b**) N/P = 2.0 and PnBA_39_-*b*-PNIPAM_87_-*b*-QPDMAEA_35_/DNA polyplexes at (**c**) N/P = 1.0 and (**d**) N/P = 2.0.

Figure 5 shows the dependence of the scattering intensity and hydrodynamic diameter of the terpolymer/DNA polyplexes on temperature. A constant increase in the scattering intensity (increase in the mass of the particles) is seen for PnBA_39_-*b*-PNIPAM_87_-*b*-PDMAEA_35_/DNA polyplexes at N/P = 1 (Figure 5a) and up to 45 °C. In the range of 45–55 °C, a plateau is reached. The R_h_ follows a similar pattern and increases as the temperature rises up to 35 °C, remains practically unchanged between 35 and 50 °C and increases again from 50 to 55 °C. This behavior shows that as the temperature increases, which means that PNIPAM blocks becomes less hydrophilic and shrink, the polyplexes tend to form larger aggregates. Importantly, the response of the terpolymers to temperature variations is maintained after their complexation with the DNA. Similar behavior is observed for N/P = 2 (Figure 5b).

For the quaternized terpolymers/DNA polyplexes at N/P = 1 (Figure 5c), an increase can be observed in the scattering intensity from 35 °C and above, but at a much lower scale than at PnBA_39_-*b*-PNIPAM_87_-*b*-PDMAEA_35_/DNA polyplexes, while the changes in R_h_ are insignificant. This means that as the temperature increases and the PNIPAM block shrinks, disassociation of the aggregates (if they exist) or shrinkage of the polymeric micelles may take place. Similar results are obtained for N/P = 2, with the changes in the size of the polyplexes being slightly larger, indicating that the polymer/DNA ratio is a very important factor regarding the response of the polyplexes to temperature variations.

**Figure 6:**
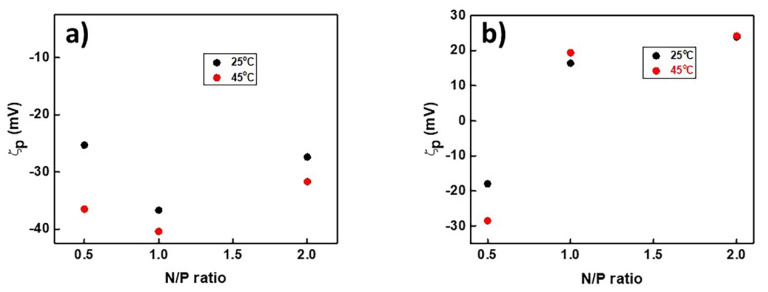
Variation in ζ-potential with N/P ratio for PnBA_39_-*b*-PNIPAM_87_-*b*-PDMAEA_35_/DNA (**a**) and PnBA_39_-*b*-PNIPAM_87_-*b*-QPDMAEA_35_/DNA (**b**) polyplexes at 25 °C and at 45 °C.

The surface charge of the polyplexes, at all N/P ratios, was investigated by electrophoretic light scattering measurements at 25 °C and at 45 °C (Figure 6). For PnBA_39_-*b*-PNIPAM_87_-*b*-PDMAEA_35_/DNA at 25 °C (Figure 6a), the ζ-potential values are negative at all N/P ratios, meaning that there is uncomplexed DNA in the solution. The explanation behind this observation is that the terpolymers are partially protonated and, for this reason, the electrostatic interactions are weak. At 45 °C, a temperature above the LCST of PNIPAM, the largest change is observed at N/P = 0.5, where the solution is not colloidally stable. For the remaining N/P ratios, there were no important changes.

As far as the PnBA_39_-*b*-PNIPAM_87_-*b*-QPDMAEA_35_/DNA polyplexes at 25 °C are concerned (Figure 6b), the ζ-potential values are negative for N/P = 0.5, due to the existence of free DNA in the solution. At N/P = 1 and N/P = 2 ratios, where the quaternized amine groups of the QPDMAEA cationic block are either in equilibrium or in excess in comparison to the negatively charged phosphate groups of DNA, the ζ-potential values are positive. This may indicate either the existence of micelles that have not complexed with the DNA, or that each DNA molecule interacts with more than one micelle, creating aggregates the outer parts of which carry positive charges. The latter seems to be the most probable scenario.

**Figure 7:**
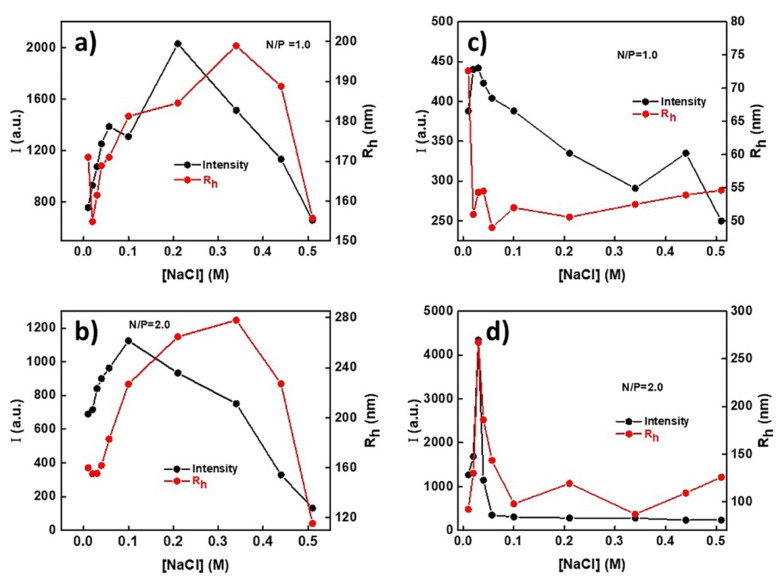
Scattering intensity and R_h_ as a function of ionic strength for PnBA_39_-*b*-PNIPAM_87_-*b*-PDMAEA_35_/DNA polyplexes at N/P = 1.0 (**a**) and N/P = 2.0 (**b**) and for PnBA_39_-*b*-PNIPAM_87_-*b*-QPDMAEA_35_ at N/P = 1.0 (**c**) and N/P = 2.0 (**d**).

The effect of ionic strength in the terpolymer/DNA polyplexes was also studied, as it is a very important parameter for the efficacy of a cationic polymer as nonviral gene delivery system. Figure 7 depicts the changes in the scattering intensity and hydrodynamic diameter of the polyplexes as a function of ionic strength at N/P = 1 and N/P = 2 ratios.

For PnBA_39_-*b*-PNIPAM_87_-*b*-PDMAEA_35_/DNA polyplexes at N/P = 1 (Figure 7a), the scattering intensity increases C_NaCl_ = 0.2 M and decreases for higher salt concentrations. The initial increase is translated into an increase in the mass of the polyplexes, which continues until their disintegration, where the decrease in the scattering intensity begins, showing that the polyplexes are not stable at higher salt concentrations. The R_h_ of the polyplexes exhibits a similar trend. It increases till C_NaCl_ = 0.32 M and decreases thereafter. A similar pattern is observed for the polyplexes formed at N/P = 2 (Figure 7b).

PnBA_39_-*b*-PNIPAM_87_-*b*-QPDMAEA_35_/DNA polyplexes exhibit different behavior as the salt concentration increases. At the ratio N/P = 1 (Figure 7c), the scattering intensity decreases in all cases, but at a much lower rate than the PnBA_39_-*b*-PNIPAM_87_-*b*-PDMAEA_35_/DNA polyplexes. This is also translated into the disassociation of the complexes and they are not stable upon an ionic strength increase. The R_h_ decreases from approx. 72 nm to approx. 52 nm with the first addition of salt and remains practically unchanged within the remaining ionic strength range studied.

At N/P = 2 (Figure 7d), an initial increase is observed in the scattering intensity till C_NaCl_ = 0.04 M, followed by a significant decrease till C_NaCl_ = 0.065 M, and the intensity remains constant at higher ionic strength values. The R_h_ behavior follows the same pattern as the scattering intensity. This shows that dissociation of the polyplexes takes place even at relatively low salt concentrations.

**Scheme 2:**
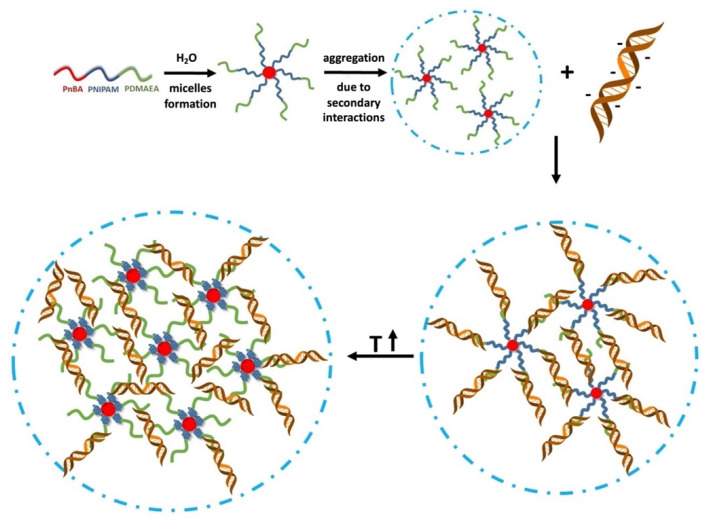
Schematic illustration of the complexation process of PnBA-b-PNIPAM-b-(Q)PDMAEA triblock terpolymers with DNA.

After evaluation of all the results obtained from the physicochemical characterization of the terpolymer/DNA complexes, the complexation process is illustrated in Scheme 2. The effect of temperature is much more visible in the case of PnBA-*b*-PNIPAM-*b*-PDMAEA/DNA complexes, where the electrostatic interactions are weaker.

### 3.2. Encapsulation of CUR in the PnBA-b-PNIPAM-b-PDMAEA Polymeric Micellar Aggregates

**Figure 8:**
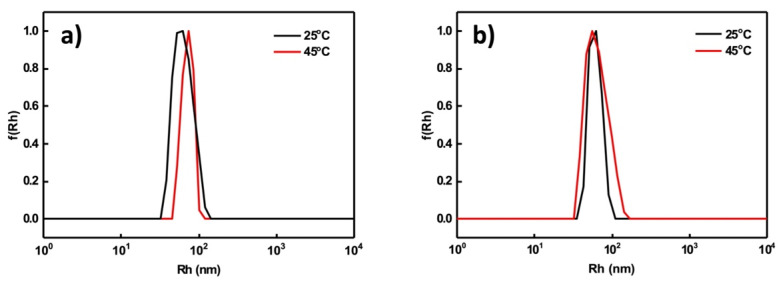
Comparative size distributions graphs from DLS measurements for CUR-loaded PnBA_39_-*b*-PNIPAM_87_-*b*-PDMAEA_35_ micelles as a function of temperature for 10% CUR (**a**) and 20% wt CUR (**b**).

The capability of PnBA-b-PNIPAM-b-PDMAEA terpolymers to act as drug delivery carriers was also investigated by encapsulating curcumin (a model hydrophobic drug). Curcumin is considered an anticancer and anti-inflammatory drug with increased hydrophobicity [41,42]. Dynamic light scattering and fluorescence spectroscopy measurements were carried out in order to determine the successful encapsulation of curcumin in the polymeric micelles and to specify the properties that curcumin imparts to the polymeric system. CUR-loaded micellar aggregates with 10% and 20% *w/w* targeted entrapment levels (relative to the hydrophobic PnBA content) for PnBA_39_-*b*-PNIPAM_87_-*b*-PDMAEA_35_ copolymer were prepared according to the protocol described above. UV–Vis determinations, utilizing a calibration curve, showed that the 10% *w/w* formulation actually resulted in 27% drug loading efficiency with a drug loading of 0.7%, while for the 20% *w/w* formulation, the values were 59% and 1.5%, respectively. Despite the rather low CUR entrapment, DLS measurements of the loaded terpolymer nanostructure were recorded at 25 °C, 45 °C and at a 90° measuring angle. The fluorescence spectroscopy study was accomplished in the same temperature range. Figure 8 illustrates comparative plots of the size distributions of CUR-loaded PnBA_39_-b-PNIPAM_87_-b-PDMAEA_35_ nanostructures.

The size distributions from DLS measurements presented in Figure 8 reveal that the addition of curcumin (10 wt% and 20 wt% relative to the hydrophobic PnBA block) causes a slight decrease in the dimensions of the polymeric micelles, which is accompanied by a significant reduction in the polydispersity index. This observation proves that the entrapment of curcumin significantly improves the self-organization of the drug-loaded nanoparticles and may denote that the hydrophobic interactions created between the copolymer and the drug facilitate the effective encapsulation of CUR into the polymeric micelles. From the results shown in Table 2, it is worth noting that when the temperature was increased, a significant increase occurred in the mass of CUR-loaded nanoparticles, which indicates the formation of more compact micellar aggregates when curcumin is present in the nanosystem.

**Figure 9:**
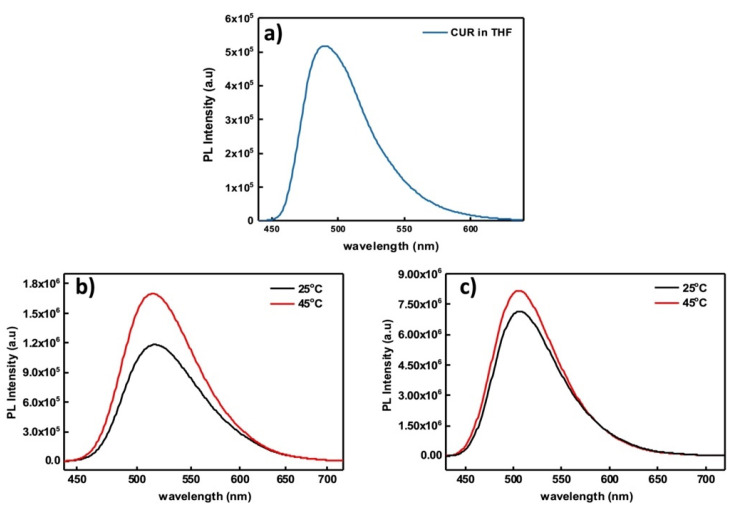
Fluorescence of CUR in THF (c_CUR_ = 100 μg mL^−1^) (**a**), fluorescence of 10 wt% (**b**) and 20 wt% (**c**) CUR-loaded PnBA_39_-*b*-PNIPAM_87_-*b*-PDMAEA_35_ micellar aggregates.

As discussed in our previous work, in addition to its beneficial properties, curcumin can also be used as an imaging agent for bioimaging applications due to the strong endogenous fluorescence that it presents [46]. Given that, fluorescence spectroscopy measurements were performed on the CUR-loaded micellar aggregates. It has been proved that the solubility of curcumin in water (4.2 μg mL^−1^) is very low [43]. However, when entrapped in the hydrophobic environment of amphiphilic copolymer self-assemblies, its solubility increases significantly. CUR-loaded terpolymer micellar aggregates displayed an important increase in curcumin solubility, by a factor of 6 (10 wt% CUR, 25 μg mL^−1^) and 12 times (20 wt% CUR, 50 μg mL^−1^) compared to the case of water. In Figure 9, it can be observed that the peak of curcumin at 489 nm in THF shifted to 514 nm (10 wt% CUR) and 504 nm (20 wt% CUR), respectively, in aqueous media. This shift is speculated to be due to the hydrophobic interactions that take place between CUR and the hydrophobic PnBA domains of the terpolymer. As far as fluorescence intensity is concerned, an interesting change occurred when temperature was increased at 45 °C. In both cases (i.e., for 10% and 20% wt CUR), a significant increase in fluorescence intensity is observed, indicating that curcumin is arranged within the hydrophobic domains of the macromolecular assemblies according to the thermo-responsive structural reorganization of the copolymer. It is observed that by increasing the solution temperature, aggregates of greater mass are formed. Thus, CUR-loaded nanoparticles become more compact, which changes entrapped CUR spatial arrangements and interactions within the aggregates and ultimately results in higher fluorescence intensity.

**Scheme 3:**
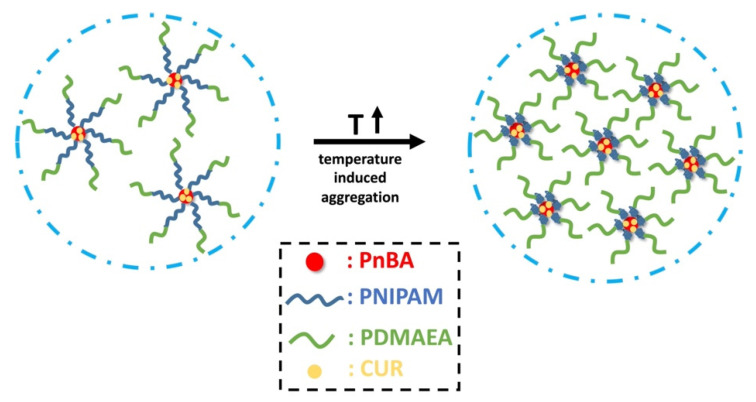
Schematic illustration of CUR-loaded micellar aggregates and the effect of temperature.

The encapsulation procedure and the effect of temperature on the CUR-loaded aggregates is depicted in Scheme 3. The observed temperature dependence of fluorescence may be of potential utilization in bioimaging, e.g., in discriminating differences in temperature within cell or tissue environments.

## 4. Conclusions

The complexation ability of PnBA-*b*-PNIPAM-*b*-PDMAEA and PnBA-*b*-PNIPAM-*b*-QPDMAEA triblock terpolymers with DNA in aqueous solutions was studied. The electrostatic interactions between the quaternized terpolymers and DNA were found to be significantly stronger than the PnBA-*b*-PNIPAM-*b*-PDMAEA/DNA ones, due to the existence of permanent positive charges. The size of the PnBA-*b*-PNIPAM-*b*-PDMAEA/DNA polyplexes is larger (aggregate formation) and they exhibit increased responses to temperature variations because of the presence of the PNIPAM block. On the contrary, the polyplexes formed between PnBA-*b*-PNIPAM-*b*-QPDMAEA and DNA are smaller due to the higher solubility of the particular terpolymers in aqueous media because of the existence of more positive charges. These polyplexes do not present significant changes with temperature variations due to their stronger polyelectrolyte character. The PnBA-*b*-PNIPAM-*b*-PDMAEA terpolymers were also tested as nanocarriers for the encapsulation of the hydrophobic model drug curcumin in the hydrophobic PnBA cores of the formed micelles. The size of the drug-encapsulated nano-assemblies was smaller than the empty ones and this difference became larger at 45 °C, above the LCST of PNIPAM. Moreover, the endogenous fluorescence of curcumin exhibits a significant increase as the temperature increases above the LCST of PNIPAM, showing that this nanosystem can be promising for potential bioimaging applications.

## Figures and Tables

**Table 1:** Molecular characteristics of PnBA-b-PNIPAM-b-(Q)PDMAEA terpolymers.

Sample	M_w_^a^ (10^−4^) (g mol^−1^)	M_w_/M_n_^a^	Composition ^b^ (% wt) PnBA/PNIPAM/(Q)PDMAEA
PnBA_39_-*b*-PNIPAM_87_-*b*-PDMAEA_17_	1.82	1.25	28/59/13
PnBA_39_-*b*-PNIPAM_87_-*b*-PDMAEA_35_	2.40	1.27	25/54/21
PnBA_39_-*b*-PNIPAM_87_-*b*-QPDMAEA_17_	2.05 ^c^	-	26/51/23
PnBA_39_-*b*-PNIPAM_87_-*b*-QPDMAEA_35_	2.90 ^c^	-	21/45/34

^a^ Determined by SEC, ^b^ Determined by ^1^H-NMR, ^c^ Calculated for 100% conversion.

**Table 2:** DLS data for free and CUR-loaded terpolymer micellar aggregates.

Sample	CUR (%)	Temperature (°C)	Intensity ^a^ (a.u.) Free/Loaded	R_h_^a^ (nm) Free/Loaded	PDI ^a^ Free/Loaded
PnBA_39_-*b*-PNIPAM_87_-*b*-PDMAEA_35_	10	25	5960/14,870	83/71	0.35/0.16
		45	19,000/30,000	54/73	0.22/0.14
	20	25	5960/17,450	83/61	0.35/0.13
		45	19,000/37,000	54/64	0.22/0.15

^a^ By DLS at 90° angle.

## Data Availability

The data presented in this study are available on request from the corresponding author.
